# Breast cancer diagnosis using frequency decomposition of surface motion of actuated breast tissue

**DOI:** 10.3389/fonc.2022.969530

**Published:** 2022-11-02

**Authors:** Jessica Fitzjohn, Cong Zhou, J. Geoffrey Chase

**Affiliations:** ^1^ Department of Mechanical Engineering, Centre for Bio-engineering, University of Canterbury, Christchurch, New Zealand; ^2^ School of Civil Aviation, Northwestern Polytechnic University, Xian, China

**Keywords:** breast cancer, screening, diagnostic, digital image elasto tomography, DIET, ROC, bootstrapping

## Abstract

This paper presents a computationally simple diagnostic algorithm for breast cancer using a non-invasive Digital Image Elasto Tomography (DIET) system. N=14 women (28 breasts, 13 cancerous) underwent a clinical trial using the DIET system following mammography diagnosis. The screening involves steady state sinusoidal vibrations applied to the free hanging breast with cameras used to capture tissue motion. Image reconstruction methods provide surface displacement data for approximately 14,000 reference points on the breast surface. The breast surface was segmented into four radial and four vertical segments. Frequency decomposition of reference point motion in each segment were compared. Segments on the same vertical band were hypothesised to have similar frequency content in healthy breasts, with significant differences indicating a tumor, based on the stiffness dependence of frequency and tumors being 4~10 times stiffer than healthy tissue. Twelve breast configurations were used to test robustness of the method. Optimal breast configuration for the 26 breasts analysed (13 cancerous, 13 healthy) resulted in 85% sensitivity and 77% specificity. Combining two opposite configurations resulted in correct diagnosis of all cancerous breasts with 100% sensitivity and 69% specificity. Bootstrapping was used to fit a smooth receiver operator characteristic (ROC) curve to compare breast configuration performance with optimal area under the curve (AUC) of 0.85. Diagnostic results show diagnostic accuracy is comparable or better than mammography, with the added benefits of DIET screening, including portability, non-invasive screening, and no breast compression, with potential to increase screening participation and equity, improving outcomes for women.

## Introduction

Breast cancer is the most frequent cancer and leading cause of cancer deaths in women worldwide (1–4). In 2018 over 620,000 breast cancer related deaths were recorded and both incidence and deaths are expected to rise (5). Early detection is associated with increased survival rates due to cancer being found at an earlier, more curable stage (3, 6). Currently, x-ray mammography is the gold standard for breast cancer screening and is estimated to reduce mortality by up to 25% (7). However, mammography remains controversial due to painful breast compression and invasive radiation exposure (1). Reduced success in women with dense breast tissue (almost 50% of women (8)), arises due to fibroglandular tissue masking the presence of tumors in mammographic images (9–11), further contributing to mammography’s radiologist-dependent performance and reducing sensitivity to as low as 27% (12). Because of these drawbacks, mammography is not recommended for younger women (13, 14), where poorer outcomes do not outweigh the risks, creating inequity of breast care for younger women.

Although mammography is accepted as a large scale screening tool, a number of issues have led mammography sensitivity to be significantly overstated (8). Hollingsworth suggests many mammography studies use a cohort inclusive of palpable tumors, which are larger and therefore expectedly easier to diagnose (15, 16). Similarly, sensitivity calculations based on prevalence screens only (the initial screen) result in a disproportionate number of larger tumors and consequently an inflated value for sensitivity (8, 17).

Further, many studies use interval cancers, cancers which occur following a negative mammogram, but before the next round of screening (18), as a measure of false negatives. This methodology is also flawed, as while some cancers may have begun and developed between screens, slower growing tumors may not present in the screening interval, and consequently would be diagnosed as true positives in the following screen, despite being missed previously. This issue results in sensitivity dependent on screening interval and higher sensitivity than studies comparing supplemental imaging modalities. This method of assessment will also impact specificity values, as false negatives in past mammograms would be included as a true negative reading. In other words, studies which exclude the use of a complementary modality assume mammography diagnosis is true until proven false with only future mammograms to compare to. Even then, cancer found in subsequent mammograms are considered true positives and assumed to have began and developed between screens. This process is clearly methodologically over-simplistic and unsound, and accuracy estimates of sensitivity or specificity using these methods should be discounted appropriately.

Digital Image Elasto Tomography (DIET) (19–23) is an alternative breast cancer screening technology. The system is portable with non-invasive testing, and thus able to increase equity for young women and those living rurally. DIET involves a patient lying face down while a mechanical actuator induces steady state sinusoidal vibration in the free-hanging breast. Five digital cameras surrounding the breast capture images of the surface vibration at different stages using synchronized strobe lights. These images are converted into displacement data for over 14,000 reference points using surface volume and optical flow techniques by Tiro Medical (Christchurch, New Zealand). Cancer diagnosis using this surface motion data is based on shear wave transmission differences reflected in the surface motion highlighting the differences in underlying tissue stiffness and damping. A significant contrast in elastic properties (400-1000%) between healthy and cancerous tissue provides a potentially highly distinguishable diagnostic, which is much greater than the 5-10% contrast in radio density used by mammography (24, 25). Thus, the DIET system detects and localises a tumor location based on identification of the higher underlying tissue stiffness, which is very different to typical internal imaging modalities.

Analysis of this data has showed potential for diagnostic success including Zhou et al’s study on hysteresis loop analysis (HLA) (26, 27) and Kashif et al’s study on modal analysis (22). Most diagnostic methods developed were exclusively tested on silicone phantom data, used in early technology development (28). Despite sophisticated silicone phantom design (23, 29), silicone phantom breasts with stiffer inclusions cannot truly imitate the inhomogenous fibrous structure of breast tissue nor the complex interactions between tumors and healthy tissue. Zhou et al’s HLA study underwent limited testing with clinical data (3 subjects) (27), but sensitivity was not high and dependent on selection of actuator input frequency for different subjects, showing inconsistent performance.

This author presented one diagnostic method using DIET displacement data, which was validated on 26 breasts (30). This method involved fitting a viscous damping model (VDM) to viscous damping distribution in different breast segments and comparing model coefficients. One model coefficient, related to stiffness showed diagnostic insight with optimal sensitivity and specificity of 77%, using clinical data.

This paper describes a fully automated, computationally efficient diagnostic algorithm, which uses displacement data from a novel 3D surface motion reconstruction DIET technology. The proposed method is based on the hypothesis stiff tumors will affect response frequencies in the breast compared to other regions containing healthy tissue, thus enabling transformation of dynamic response into a novel diagnostic metric to identify regions of higher stiffness for cancer diagnosis. This diagnostic uses a combination of frequency components analysis, surface segmentation and bootstrapping techniques, while previous diagnostic methods based on DIETs technology mainly identify damping, stiffness and modal distribution (22, 27, 30). In addition, it provides an unbiased diagnostic criteria to ensure each breast to be diagnosed independently, regardless of varying breast properties across the population, which is critical for improving the equity of screening with this technology. Overall, this work offers a novel approach to implement automated unbiased tumor detection in this DIET screening technology.

## Method

### Clinical data

Fourteen women (P1-P14) were recruited to undergo testing using a prototype DIET system, as part of a clinical trial run at Canterbury Breastcare (Christchurch, New Zealand). Thirteen women had a tumor in one breast and one women had two healthy breasts resulting in a total of 13 cancerous, 15 healthy breasts. Patient P6 also has an additional non-malignant cyst in their right (healthy) breast. Each woman underwent mammography screening prior to testing using the DIET prototype and diagnostic capabilities of the algorithm presented in this paper aim to correctly distinguish between healthy and cancerous breasts in this cohort and match the diagnostic given by mammography. Ethics approval for the experimental tests, data collection, and analysis of this data was granted by the NZ National Health and Disability Ethics Committee, South Island Regional Committee.

**Table 1** shows the patient age, tumor size and location from mammography reports for each patient with a cancerous breast, as well as the approximate breast volume of the cancerous breast calculated using the DIET measured displacement data. Tumor sizes ranged from 7 to 48mm and displacement data was available for a range of testing frequencies (20~50 Hz). While clinical data is limited to 28 breasts, the variation in breast properties and tumor sizes is large, providing a varied cohort. Ensuring diagnostic performance is robust to these variations in tumor size and breast properties is a particular focus in this paper. The accuracy of the size and location vary and can be difficult to determine from mammography images. Thus, location and size data were treated as approximate, and algorithm success focused on correct diagnosis, rather than precise tumor localisation.

**Table 1:** Patient age, breast volume, tumor size and locations cancerous breasts where 12 o’clock is the top of the breast.

Subject Number	Age	Cancerous Breast	Tumor Location (around breast)	Tumor diameter (mm)	Tumor depth (mm)	Cancerous breast Volume (*cm* ^3^)
*P1*	50	Left	10 o’clock	18	35.1	691.7
*P2*	58	Left	2.30 o’clock	15	–	307.9
*P3*	58	Right	10 o’clock	14	51.7	265.8
*P4*	37	Left	3-5 o’clock	48	–	740.8
*P5*	38	Left	12.30 o’clock	14	26.3	239.5
*P6*	45	Left	6 o’clock	12	13.3	708.6
*P7*	55	Left	2 o’clock	23	78.8	673.7
*P8*	51	Right	10.30 o’clock	37	83.9	1022.0
*P9*	55	Left	12 o’clock	16	53.1	474.4
*P10*	50	Left	9-12 o’clock	7	–	1057.0
*P11*	51	Right	10 o’clock	7	–	342.9
*P12*	55	Left	11 o’clock	10	–	444.2
*P13*	47	Right	9-3 o’clock	18	–	593.8

It is important to note patient P14, with two healthy breasts, originally had their right breast, P14R, diagnosed as cancerous, which was later discovered to be healthy tissue. This result shows a false positive in mammography and correct diagnosis of this breast using DIET would further demonstrate its diagnostic potential. Occasionally, difficulties in optical flow or image reconstruction resulted in a lack of data for some subjects or a limited number of available input frequencies. The algorithm presented in this paper uses low actuator input frequencies, resulting in the exclusion of two healthy breasts in P4 and P13. The result is 13 cancerous and 13 healthy breasts used in this analysis and the diagnostic algorithm presented in this paper aims to correctly diagnose these subjects.

### Diagnostic criteria

As mentioned, sensitivity and specificity of mammography has been overstated in many studies. To assess approximate true sensitivity and specificity, studies were considered if diagnostic results of mammography were validated using another modality, such as ultrasound or MRI. Values for both dense and non-dense breasts were used when studies distinguished between the two, based on approximately 50% of women having dense breasts (8). Average sensitivity of the ten studies assessed was 60% (40%-78% range) and average specificity was 80% (46%-99% range) (31–40). These accuracy values are more suitable for comparing mammography to other breast screening modalities.

The area under a receiver operator characteristic (ROC) curve (AUC) between 0 and 1 is commonly used to compare diagnostic methods, as a higher AUC indicates a better optimal sensitivity and specificity. AUC greater than 0.7, 0.8 and 0.9 are considered acceptable, excellent and outstanding, respectively (41). The average ROC curve AUC value for mammography across eight studies was 0.73 (0.54-0.84 range), which will also be used to compare diagnostic success of the method presented in this paper (12, 31, 40, 42–46).

Two overall accuracy criteria are suggested to show diagnostic potential for the algorithm presented:

1. *Diagnostic sensitivity and specificity similar to mammography (60% sensitivity, 80% specificity)* Achieving comparable diagnostic accuracy to mammography would allow the DIET technology to realise its many benefits including comfort, portability and safety for all women without compromising on diagnostic success.
2. *A highly sensitive diagnostic algorithm is achieved (80% sensitivity, 65% specificity)* An algorithm capable of providing sensitivity higher than mammography will be considered a success even if specificity is slightly lower. This criteria is due to the ease of DIET testing and its many other benefits making it an attractive solution for breast screening. There is potential for added clinical breast exam (CBE) or other breast screening technologies with higher specificity to optimise diagnosis and reduce false positive biopsies, following a highly sensitive diagnosis using DIET.

To maximise the benefit of the DIET technology, this diagnostic algorithm should meet the criteria in **Table 2**.

**Table 2:** Diagnostic criteria to assess the success of diagnostic algorithms using DIET.

1. Unbiased diagnostic, unaltered by known tumor identification or symptoms
2. Full automation with no human interpretation of results required
3. Ability to diagnose tumors down to 7mm, the smallest tumors in this clinical data
4. Robust to varying breast sizes and densities
5. Have AUC greater than mammography (>0.73)
6. Meet one of the following accuracy criteria:
a. Sensitivity (60%) and specificity (80%) similar to mammography
b. Sensitivity (80%) and specificity (65%) highly sensitive screening tool

### Stiffness dependent vibration

The stiffness dependence of vibration frequency is well documented (47). Stiffer materials vibrate at higher frequencies, based on:


(1) $$\omega =\sqrt{\frac{k}{m}}$$

where *ω* is frequency, *k* is stiffness and *m* is mass. Thus, the presence of tumors, known to be 4~10 times stiffer than healthy tissue [24,48,49,50], may result in a visible increase in response frequency, given mass at a local point is similar, yielding:


(2) $$\omega_{tumor}=\sqrt{\frac{k_{tumor}}{m}}=\sqrt{\frac{(4∼10)\;k_{healthy}}{m}}=2∼3.16\;\omega_{healthy}$$

This equation further suggests higher frequency response in cancerous breasts, or breast segments, of a magnitude 2~3 times the response of healthy tissue. Development of a diagnostic algorithm using DIET concluded variation of breast properties, including stiffness and viscous damping, across the population can exceed variations between cancerous and healthy tissue in an individual (30). Therefore, it is important to understand single diagnostic thresholds for response frequencies may not be suitable for diagnosis across a varied cohort and may cause women with naturally stiff breasts to experience a disproportionate number of false positive diagnoses. The author’s prior work presented a breast segmentation methodology to analyse tissue properties in different regions of a breast (30). Healthy breasts were hypothesised to have similar breast properties in different segments and larger discrepancies were indicative of a tumor. Applying this methodology, it is hypothesised frequency response of different areas in a healthy breast will be similar; in contrast, tumor presence is expected to affect response frequency.

### Frequency component of each reference point vibration response

Displacement data for over 14,000 reference points on each breast surface was provided by Tiro Medical (Christchurch, New Zealand) following clinical testing using the DIET system at each input frequency for the 26 breasts from 14 patients in Section 2.1. The Fourier transform of each reference point signal was implemented in *Matlab* (48) to obtain frequency components of each reference point vibration. Magnitudes of frequency components were ordered, with the dominant frequency expected to be equivalent to the actuator input frequency at the induced steady state response. Based on the knowledge of high mechanical stiffness resulting in higher frequency of response, and cancerous tissue resulting in 400~1000% higher stiffness than healthy tissue, the second dominant frequency has the potential to provide diagnostic information. This latter frequency is hypothesised to be higher, but, more importantly, different in regions of the breast containing a tumor.

The second dominant frequency and its signal magnitude were obtained for each reference point. Reference points with second frequency magnitudes less than 15% of the dominant frequency magnitude were discarded to avoid using reference points where frequency composition was highly varied and the second frequency not considered particularly dominant.

Frequency composition is considered irregular when the dominant frequency is not equivalent to the input frequency. One reason for this irregularity is noise near the actuator or chest wall, the latter potentially due to issues of wave reflection. This issue was removed by removing the top and bottom 5% of points, as these areas tend to result in the most noise in both cancerous and healthy breasts (30). While necessary to remove these irregularities, acknowledging they are likely to be a source of increased false positives, it is important to limit the number of points discarded, to avoid tumors close to the nipple or chest wall being missed. Tumors developing near the chest wall are also an existing challenge in mammography due to difficulties in obtaining sufficient breast compression close to the chest wall to allow x-ray penetration (49).

**Figure 1:**
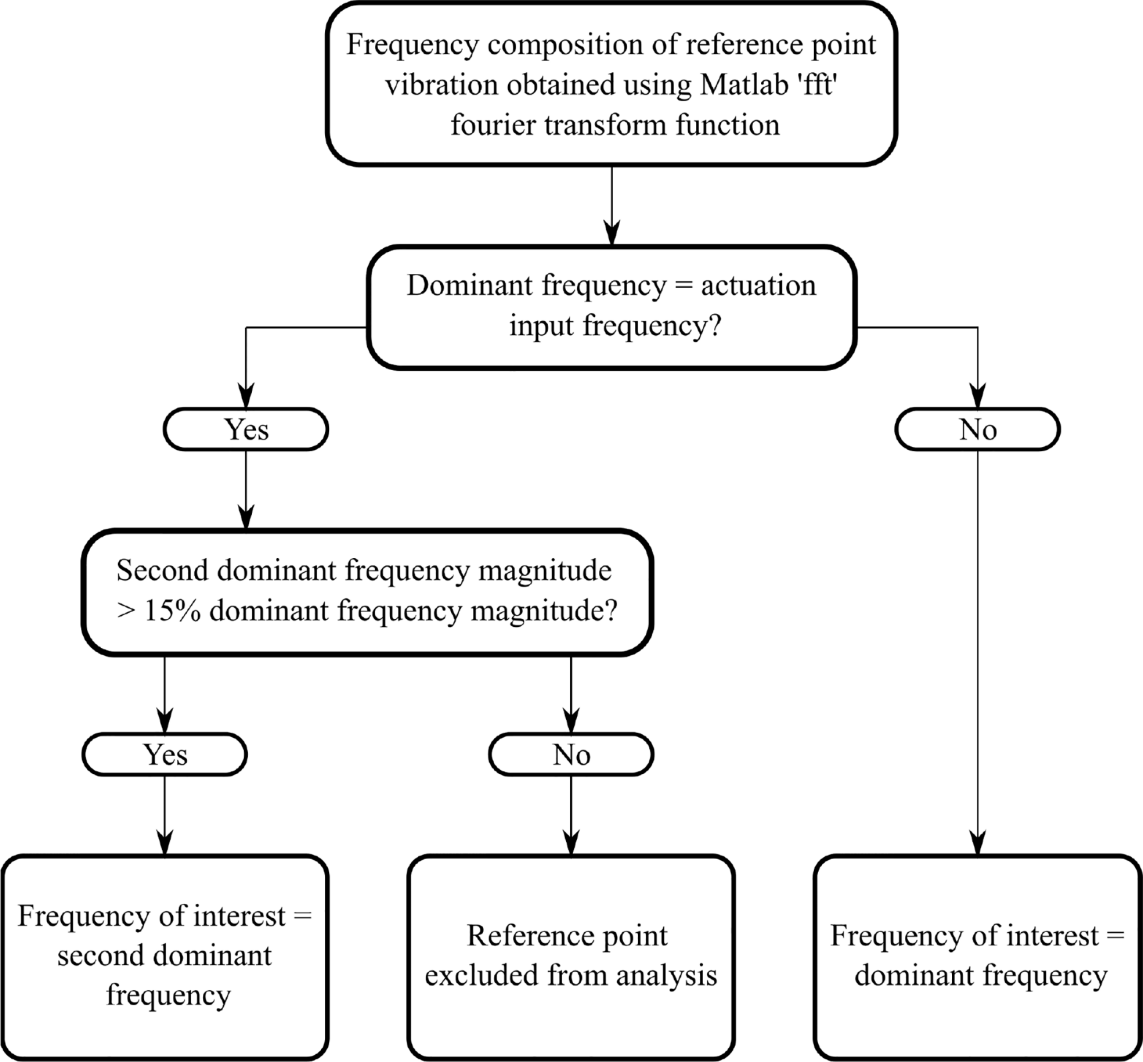
Flowchart showing method to obtain frequency of interest for each reference point.

Furthermore, irregular frequency composition could be the result of irregular vibrations for points centred near breast concavities, which are unable to be consistently removed in the current DIET image processing. Alternatively, and most importantly, it could be a result of highly variable breast tissue properties, such as stiff cancerous lesions, having a significantly large effect on frequency composition, showing high diagnostic potential. For the latter reason, reference points with dominant frequency not equal to input frequency were not excluded, and, in these cases, the dominant frequency, rather than the second dominant frequency is used as the frequency of interest. The result is a *frequency of interest* for each reference point. **Figure 1** shows a flowchart of this selection process.

Because reference points with dominant frequency not equal to input frequency could be the result of noise and breast concavities, it was necessary to manage the number used in analysis. At higher input frequencies, average dominant frequencies in segments of the breast differed to the input frequency (>2 Hz difference), suggesting high numbers of irregular points. Thus, displacement data from lower actuation input frequencies was used (20 to 23Hz) in this analysis, as they resulted in less than 10% of segments exhibiting this irregular trend, compared with 11-39% of segments in frequencies above 23 Hz.

### Breast segmentation and unbiased diagnostic criteria

Three-dimensional (3D) colour plots of the breast showing frequencies of interest are presented for three subjects at an input frequency of 23 Hz to show more regions of high response frequencies in cancerous breasts, demonstrating how comparison of these values could provide useful diagnostic insight.

**Figure 2:**
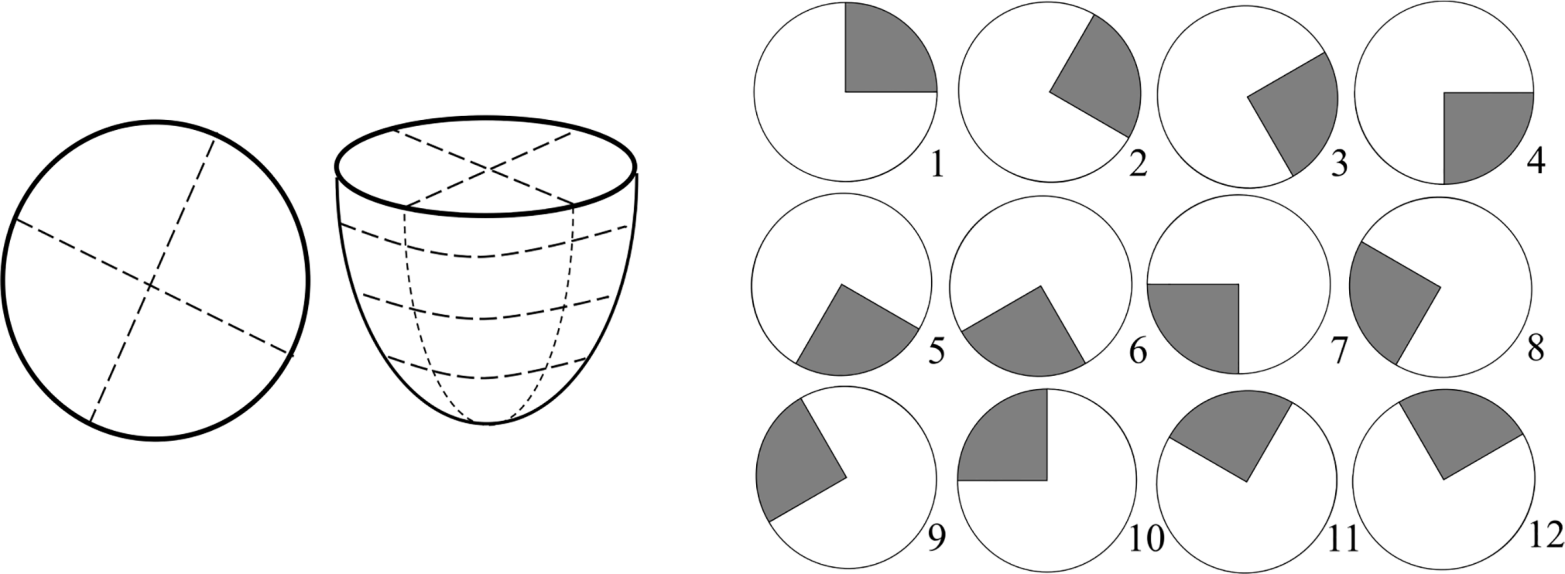
Schematic of breast segmentation including vertical (z) segmentation into four bands for a total of 16 segments (left) and diagram of 12 control segment configurations for left breast used to test robustness (right).

To implement an unbiased diagnostic algorithm utilising this *frequency of interest*, the breast was segmented into four radial segments and four vertical (z) segments, a total of 16 segments (**Figure 2**). Frequencies of interest were averaged for all reference points in each segment and mean values averaged across available frequencies from 20-23 Hz. Each z-band was analysed separately and one of the four radial segments identified as the control. The mean frequency of interest for this control segment was plotted against the mean frequencies of interest for the three other segments in the same z-band. This process was repeated for each z-band, resulting in a total of 12 data points per breast. Occasionally, all reference points in a segment were excluded, based on insufficient magnitude of the second dominant frequency (**Figure 1**), resulting in less data points per subject.

**Figure 3:**
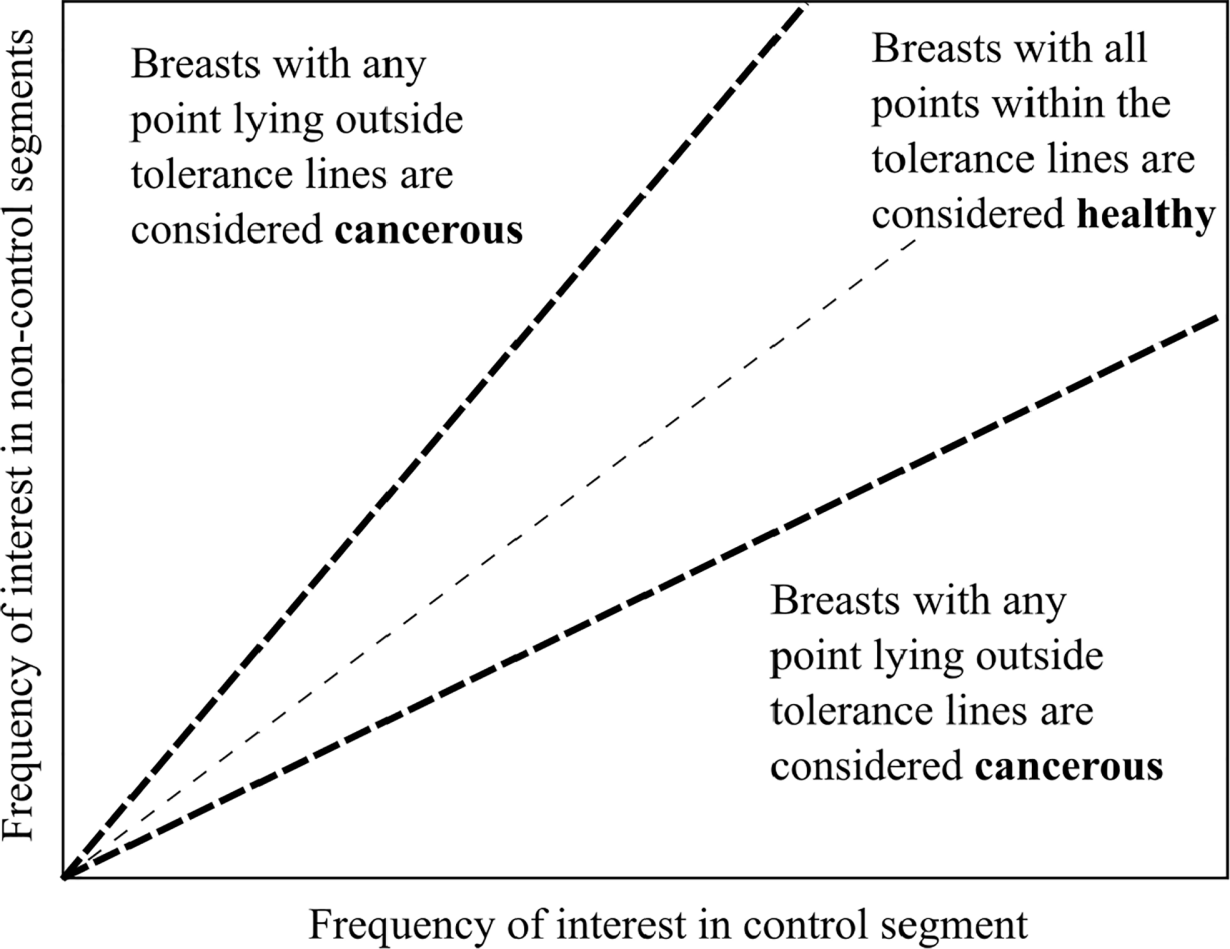
Tumor acceptance criteria showing simple diagnostic method.

Different percentage tolerances were used to analyse the degree of similarity between these averaged response frequencies in each separate z-band. Healthy breasts are hypothesised to have similar response frequencies remaining within the tolerance. In contrast, the presence of any one segment outside tolerance levels suggests a cancerous diagnosis. This diagnostic criteria is shown in **Figure 3**. To test robustness to tumor location 12 breast configurations were trialled with different control segments. **Figure 2** shows these 12 control segment configurations for the left breast. The right breast segments were the inverse of these configurations to compare outer and inner breast properties, consistently.

This segmentation process provides a method for each breast to be diagnosed independently, removing issues of highly variable breast properties across the population, within breasts of the same women and due to breast changes over time (50–55). Segmentation both radially and vertically is expected to improve diagnostic outcomes for smaller tumors, whose properties may be more easily distinguishable in a smaller segment.

### ROC curve and bootstrapping

ROC curves presenting different percentage tolerances were used to test the sensitivity of this method to predefined diagnostic tolerance levels and assess whether the criteria outlined in **Table 2** could be met. ROC curves were used to find both optimal breast configuration and tolerances, which result in criteria being met for sensitivity and specificity of this method. The discrete ROC curve for two opposite breast configurations are shown, as well as bootstrapped curves for all configurations.

**Figure 4:**
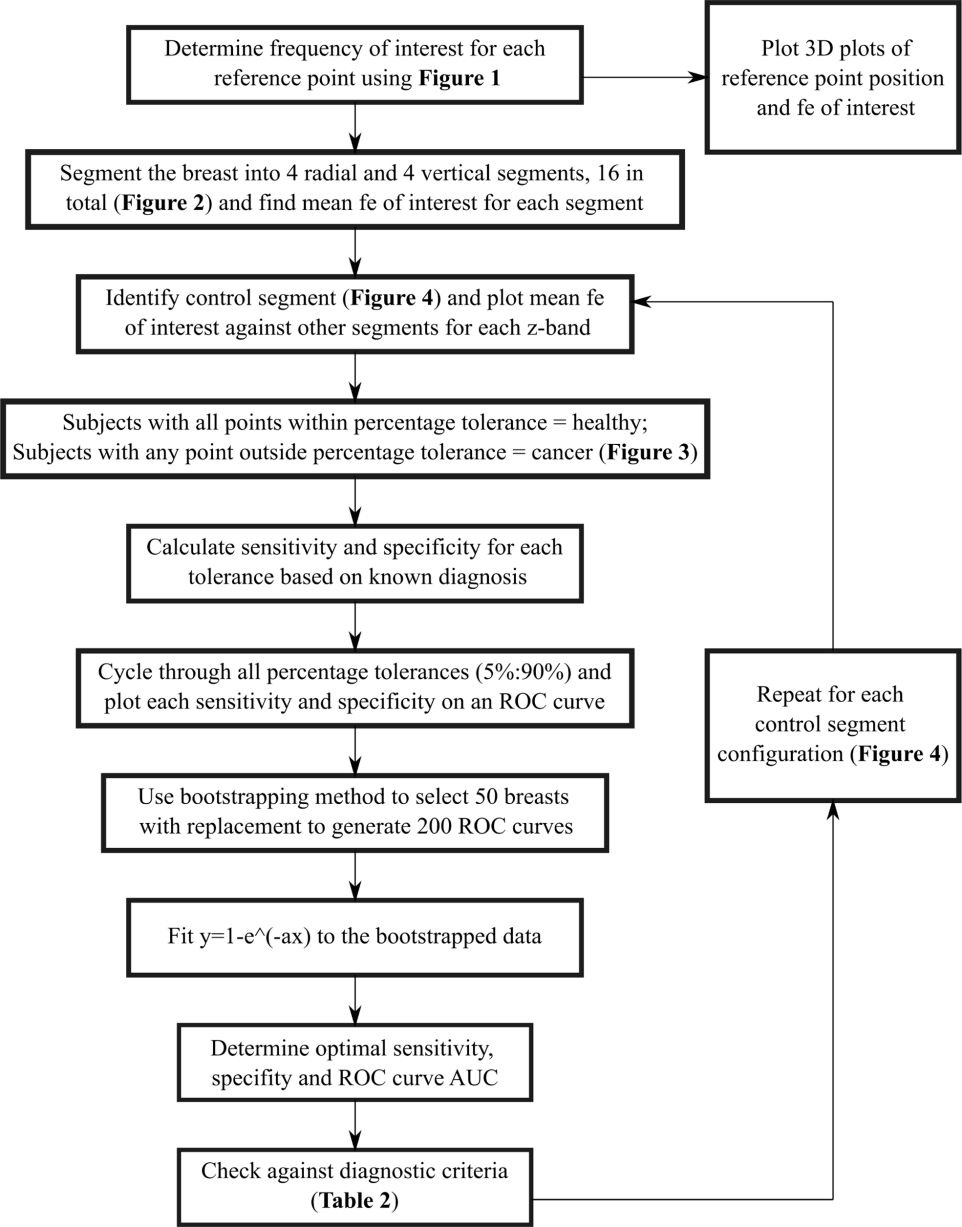
Flowchart showing full method to obtain results.

Bootstrapping is used to up-sample data and involved selecting 50 breasts with replacement from the 26 breast cohort. This selection was repeated 200 times and the varying sensitivity and specificity recorded for a number of percentage tolerance thresholds for each trial. A line of best fit was fit to the compounded points of every trial using *y=*1*-e^-ax^* using total least squares. This equation form is able to capture the linear (50:50 chance) line and, with a very large exponent, the perfect square ROC curve, as well as all likely shapes in between. It thus provides a good approximation of the diagnostic performance of each configuration in an ideally larger cohort of data and can be used to assess the performance of this algorithm against the criteria in **Table 2**. Optimal accuracy, as well as 80% sensitivity and specificity points used to assess accuracy criteria, are marked on the ROC curves. ROC curve AUC was also assessed to ensure it meets the criteria (AUC>0.73). **Figure 4** shows a flowchart of the combined methods used to generate the results presented in this paper.

The results in the following section show:

1. 3D plots showing frequency of interest for three breasts at actuator input frequency of 23 Hz, showing proof of diagnostic theory with larger discrepancies and regions of high response frequency in cancerous breasts
2. Unbiased, clinically feasible diagnosis with percentage tolerance used to determine the degree to which more dominant frequencies are different amongst segments in the same breast for both healthy and cancerous breasts for breast configurations 1 and 6
3. Identification of breast and tumor characteristics of false negative subjects
4. Identification of patient age and breast size for false negative and false positive subjects
5. A discrete ROC curve with sensitivity and specificity shown for each percentage tolerance for breast configurations 1 and 6 across all subjects
6. Smooth bootstrapped ROC curves for all breast configurations showing optimal sensitivity and specificity and points at 80% specificity and 80% sensitivity (points of interest for assessing diagnostic success against specified criteria)
7. Table outlining optimal sensitivity, specificity and assessment of each configuration against diagnostic criteria
8. Table assessing this diagnostic method against all criteria outlined in **Table 2**

## Results

### Frequency response distribution

**Figure 5:**
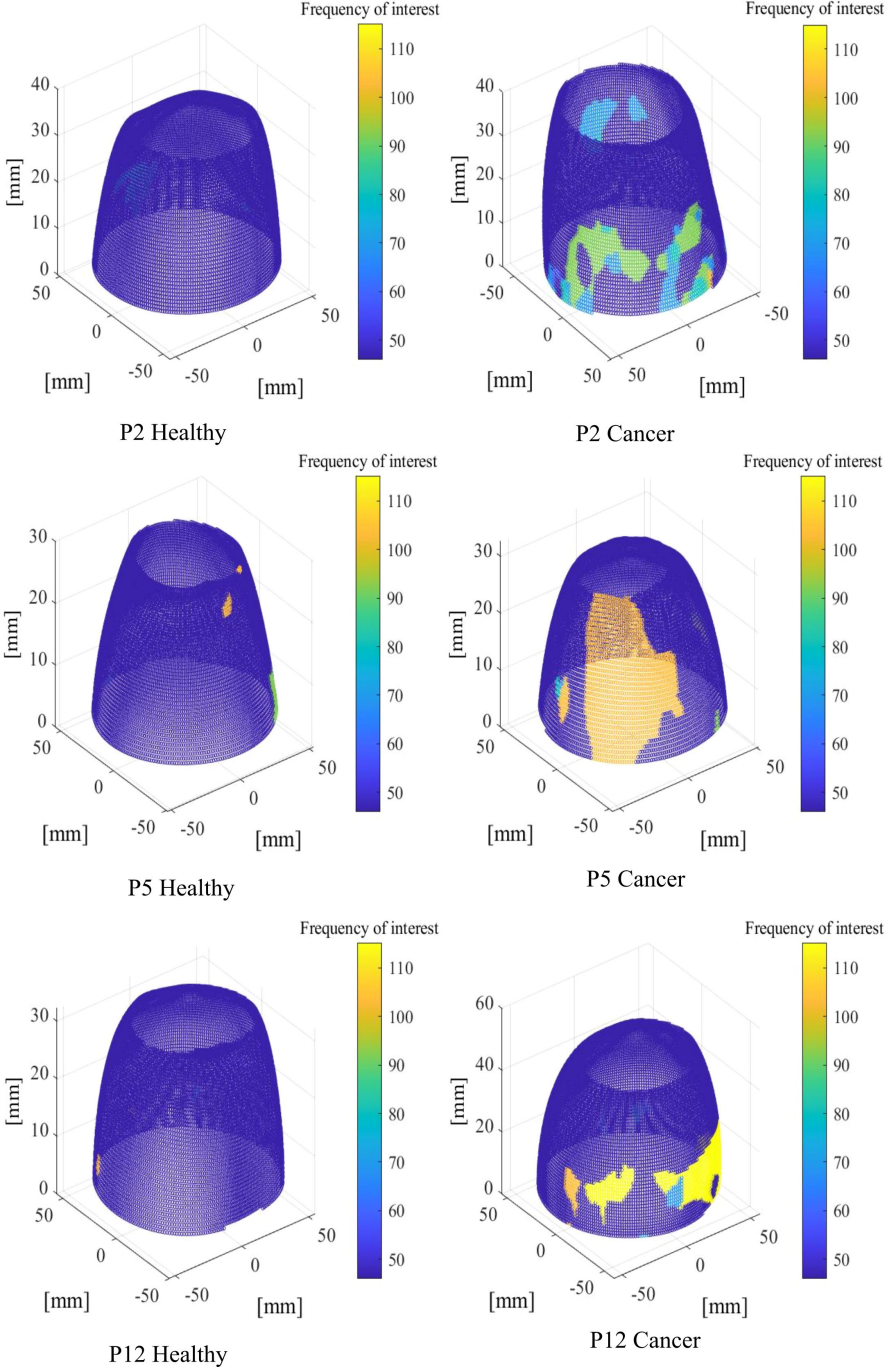
Three-dimensional (3D) plots showing areas of high frequency of interest for healthy (left) and cancerous (right) breasts at input frequency, fe=23 Hz.

**Figure 5** shows 3D plots of the distribution of frequencies of interest identified in Section 2.4 for three subjects, showing high frequencies, twice the input frequency and above. The plots clearly show a distinguishable difference in terms of frequency components of healthy and cancerous breasts on the left and right, respectively.

It is important to note, while these images show a significant contrast in frequency, and could potentially provide successful diagnosis based on image observation, direct image observation would result in human assessment of results and a lack of automation. Equally, such observation could be used to reinforce or check any automated diagnostic. Thus, these images show a proof-of-concept justification for using frequency composition to infer diagnosis, but require further development of unbiased, algorithm automation shown in consequent sections of this paper, to be clinically feasible.

### Unbiased, clinically feasible diagnosis

**Figure 6:**
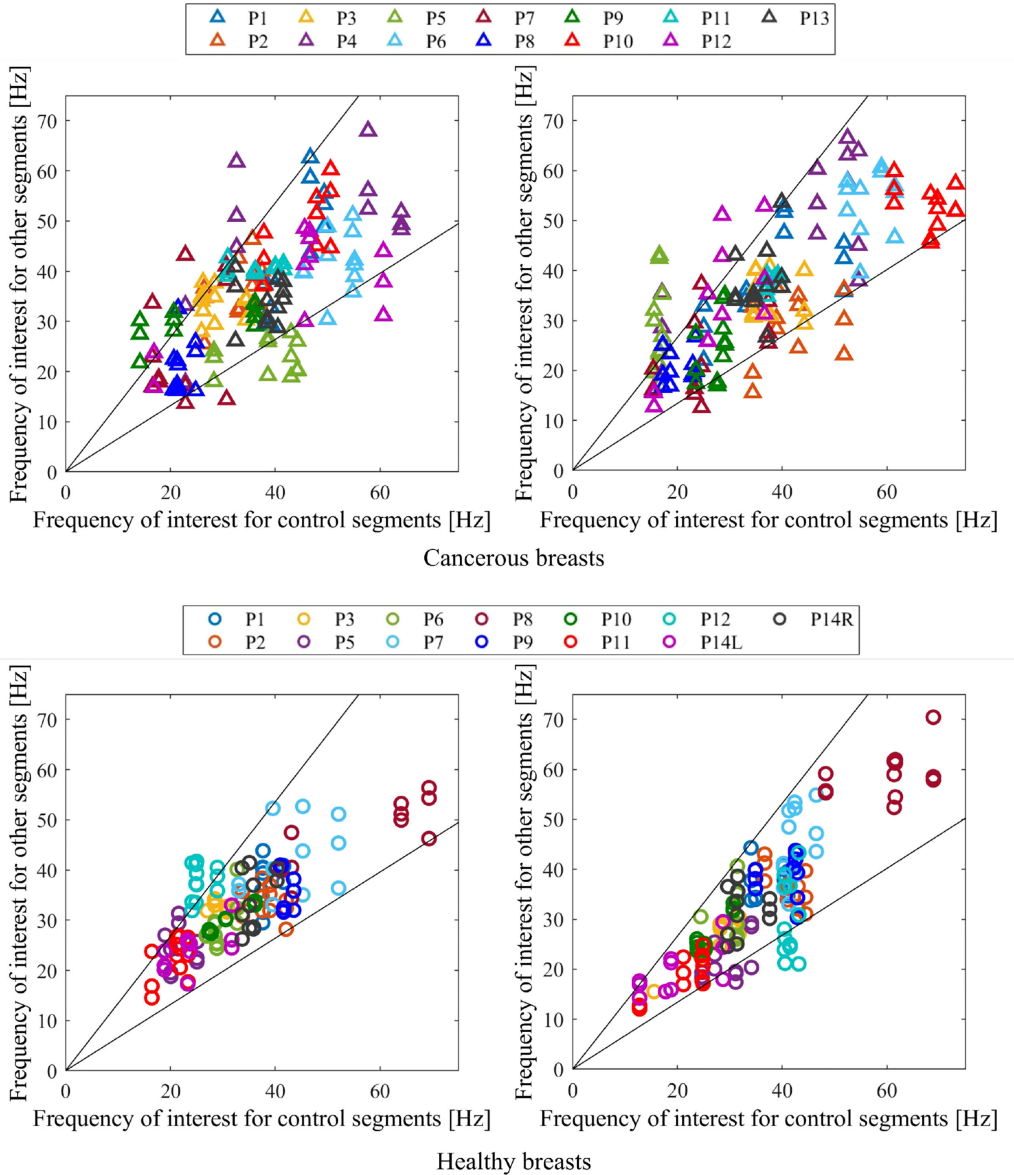
Diagnostic criteria for cancerous (top) and healthy (bottom) breasts for configuration 6 with 34% tolerance applied (left) and configuration 1 with 33% tolerance applied (right). Any one point lying outside the percentage tolerance shown results in a cancerous diagnostic.

**Figure 6** shows the diagnostic result of applying optimal percentage tolerance 34% using optimal configuration 6 and optimal percentage tolerance 33% for breast configuration 1 for both cancerous and healthy breasts. This figure shows false negatives may be dependent on configuration orientation, likely due to varying tumor locations and tumors effecting segments on either side. These two configurations, positioned in the upper outer and lower inner portions of the breast, respectively, demonstrate using the result of two separate configurations ensures all cancer is diagnosed.

**Figure 7:**
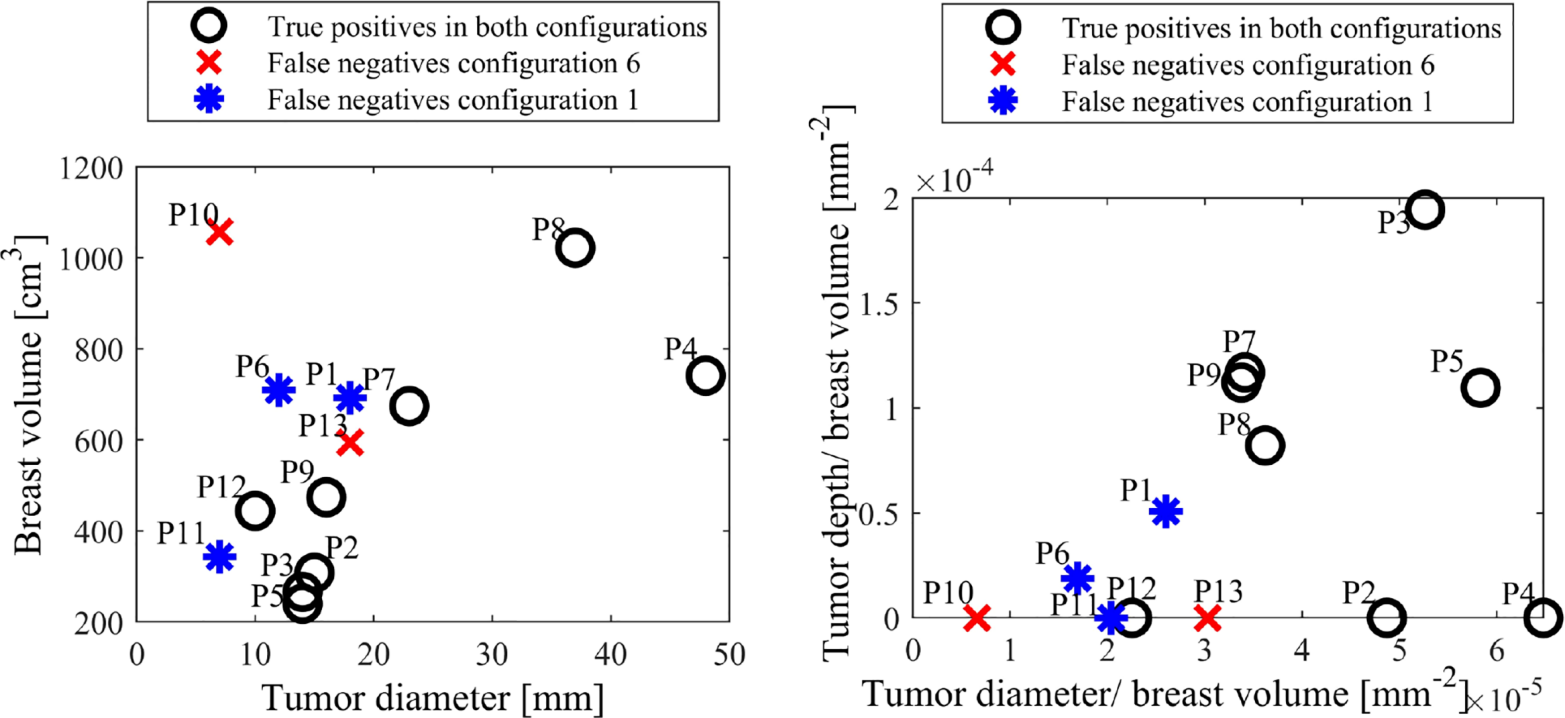
False negative tumor and breast sizes (left) and tumor depth and diameter normalised by breast volume (right) for configurations 1 and 6.

**Figure 8:**
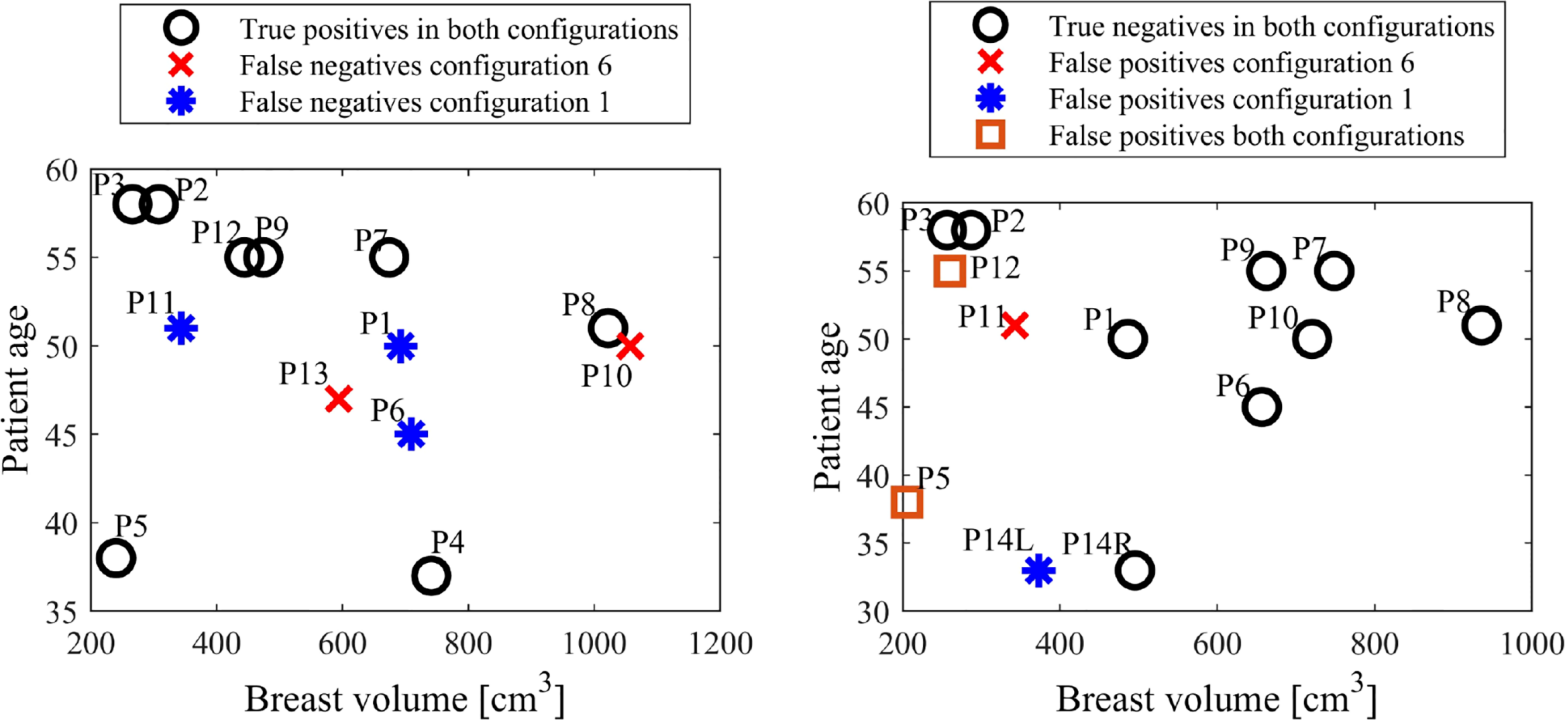
False negative (left) and false positive (right) ages and breast volumes for configurations 1 and 6.

**Figure 7** shows breast and tumor characteristics for the false negative subjects identified in configurations 1 and 6. The figures show tumor size compared to breast volume and depth (normalised by volume), respectively for each configuration. **Figure 8** shows patient age and breast size for false negatives and false positives, respectively.

**Figure 9:**
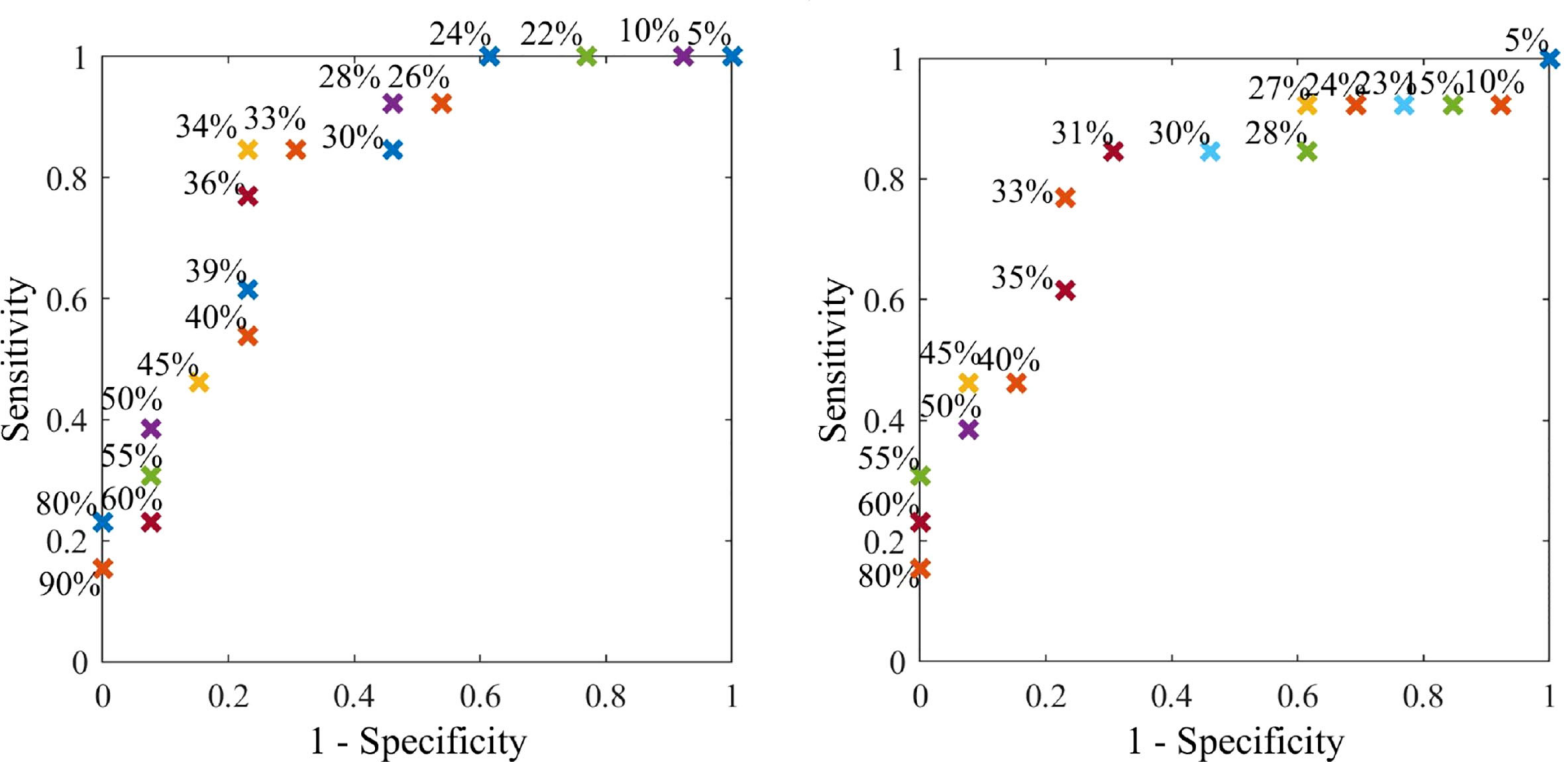
Discrete ROC curve showing diagnostic method applied at different percentage tolerances for breast configuration 6.

**Figure 10:**
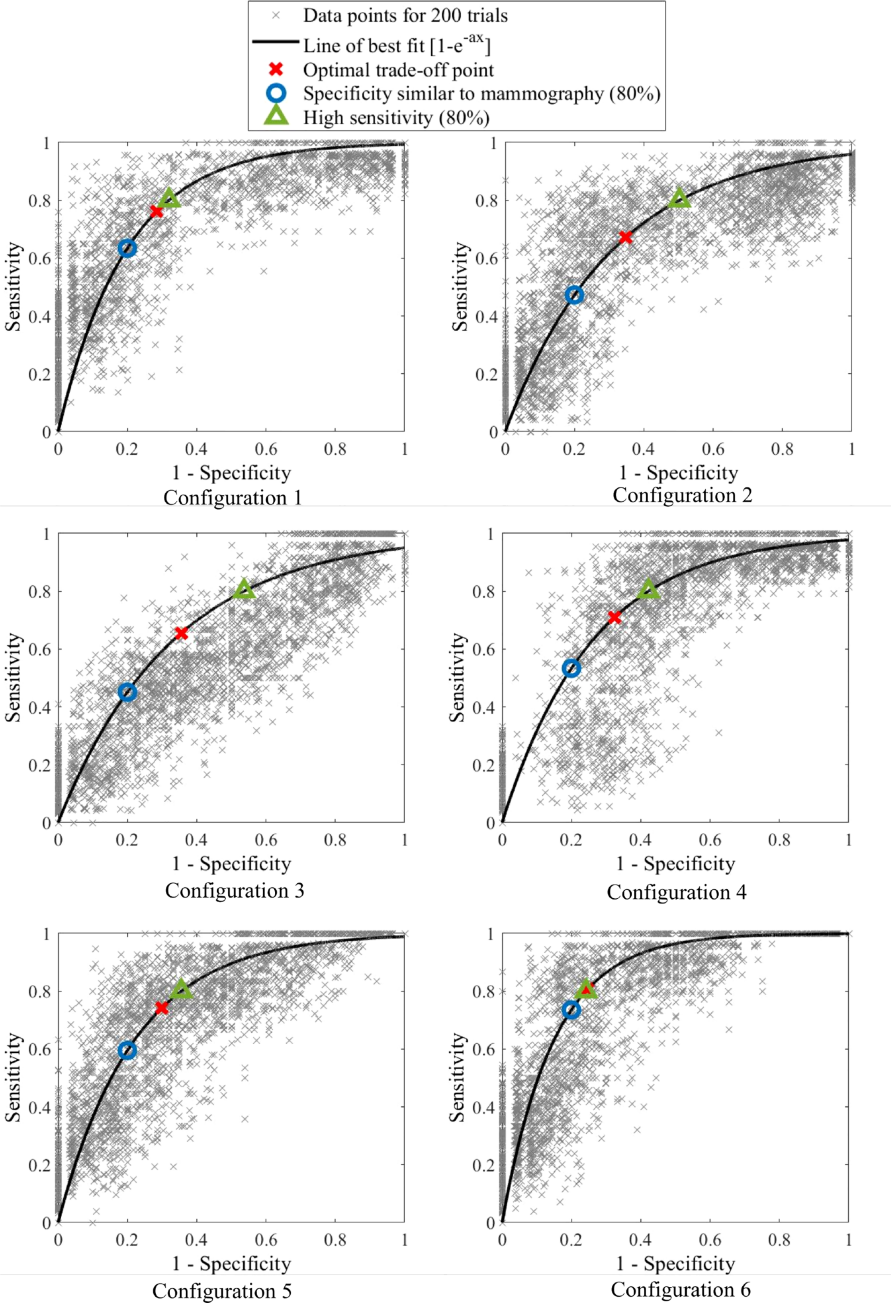
Bootstrapped ROC curves for breast configurations 1-6.

**Figure 11:**
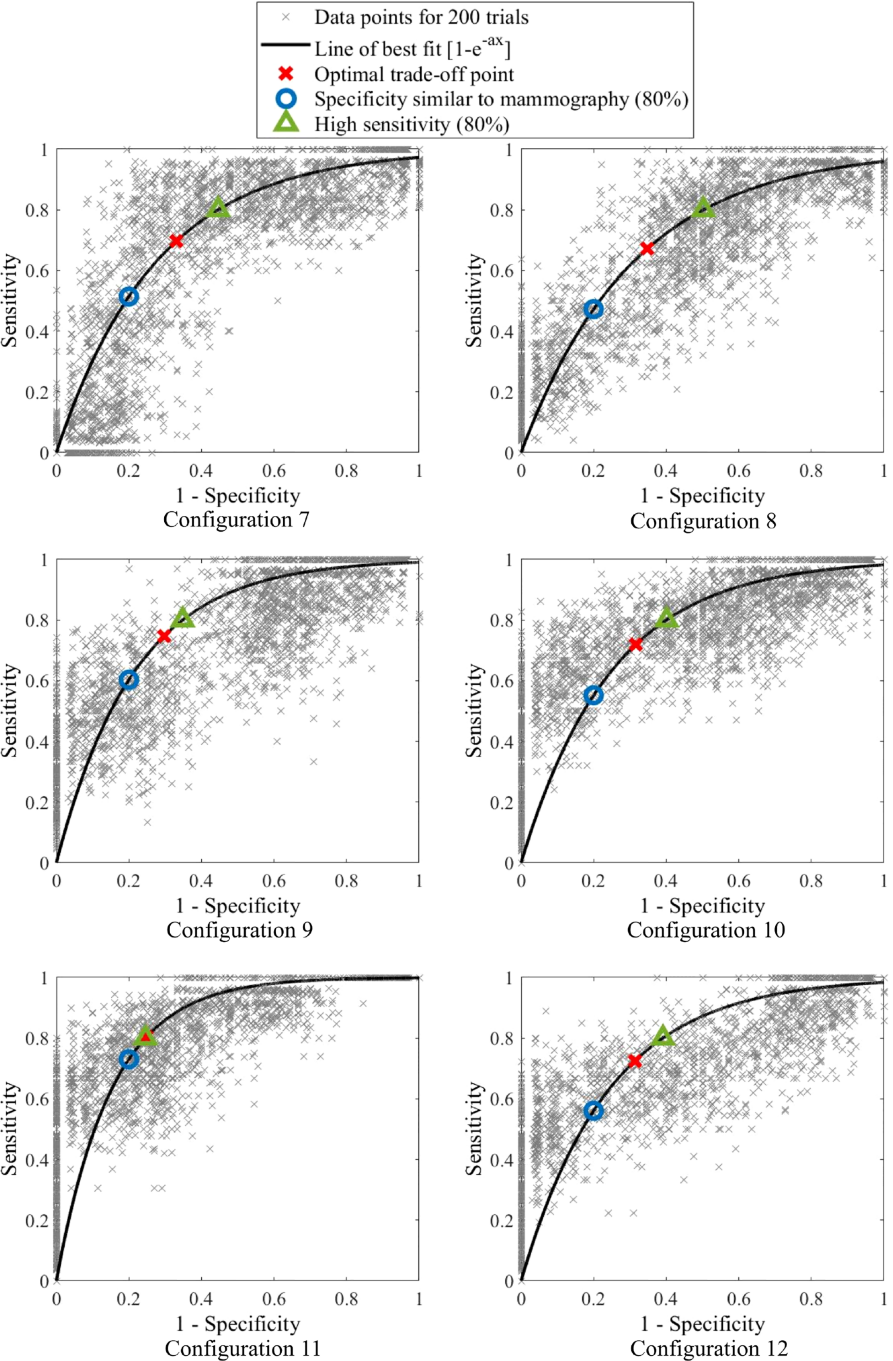
Bootstrapped ROC curves for breast configurations 7-12.

**Figure 9** shows discrete ROC curves for diagnostic performance at different percentage tolerances for configurations 6 and 1. **Figures 10**, **11** show the bootstrapped ROC curves for all breast configurations with optimal points shown and **Table 3** shows the resulting AUC and assessment against diagnostic criteria.

**Table 3:** Area under ROC curve (AUC), optimal performance point and assessment of diagnostic accuracy criteria for bootstrapping of each different breast configuration with bold values indicating the criteria is met.

Configuration	1	2	3	4	5	6	7	8	9	10	11	12
**AUC**	**0.80**	0.70	0.68	**0.74**	**0.78**	**0.85**	**0.73**	0.70	**0.79**	**0.76**	**0.85**	**0.76**
**Optimal Sensitivity**	76	67	66	71	74	81	70	67	75	72	80	72
**Optimal Specificity)**	71	65	64	68	70	75	67	65	70	68	75	69
**Sensitivity (with 80% specificity)**	**63**	47	45	53	**60**	**74**	55	47	**60**	55	**73**	56
**Specificity (with 80% sensitivity)**	**68**	50	46	58	64	**76**	62	50	**65**	60	**75**	61

The bold values show configurations, which meet the diagnostic criteria.

### Assessment against diagnostic criteria

The performance of this algorithm was assessed against the diagnostic criteria in **Table 2**, as shown in **Table 4**. All criteria were met and each 7 mm tumor was correctly diagnosed in one of the breast configurations analysed. Diagnosis using this algorithm is unbiased and completely automated and diagnostic accuracy exceeds mammography, showing significant diagnostic efficacy across this varied cohort.

**Table 4:** Assessing frequency composition method against diagnostic criteria in **Table 2**.

Criteria	Yes/No	Values
Unbiased diagnostic	Yes	
Full automation	Yes	
Ability to diagnose tumors down to 7mm	Yes	
Robust to varying breast sizes and densities	Yes	
AUC greater than mammography (>0.73)	Yes	0.85
Specificity 80%, sensitivity 60% or higher (1. similar to mammography)	Yes	74%
Sensitivity 80%, specificity 65% or higher (2. highly sensitive)	Yes	76%

## Discussion

### Proof of concept

**Figure 5** shows a visual representation of how the frequency of interest is higher and more varied in cancerous breasts. Healthy breasts generally show more areas of purple, suggesting lower frequencies and less sections of high frequency content. In contrast, cancerous breasts are generally seen to exhibit larger areas of high frequency response, which is expected based on stiffer materials vibrating at higher frequencies and cancerous tissue having stiffness 4~10 times greater than healthy tissue (24, 56–58). The examples in **Figure 5** were typical for most subjects at low frequencies (<26 Hz). Despite this significant contrast, observationof stiffness plots alone cannot quantify diagnosis, as it would fail the criteria of being automated and unbiased. These results provide proof-of-concept for the governing theory of this algorithm, but require development to prevent reliance on human interpretation, which would increase bias and error.

Furthermore, the notable differences seen here were not consistent throughout all frequencies. In general, higher frequencies (>26 Hz) resulted in much noisier and varied frequency content across all breasts, likely due to a higher incidence of wave reflection causing an increase in vibration in certain areas. As mentioned, higher frequencies tended to result in more atypical vibration with dominant frequency not equal to the input frequency in many cases. Thus, lower frequencies (20-23 Hz) were used for this frequency response analysis. Averaging the frequency of interest across the available frequencies from 20-23 Hz is unbiased and generalisable, although it should be noted, with more subject information, such as breast density, able to be found prior to screening, more optimal breast-specific testing frequencies may possibly be obtained.

### Unbiased diagnostic technique

**Figure 6** shows how this frequency of interest can be used as an indicator for cancer in an unbiased and clinically feasible way, by using a segmentation methodology and comparing frequencies of interest in different segments of a breast. **Figure 6** shows the diagnostic result for two breast configurations (1 and 6 in **Figure 2**) situated on opposite sides of the breast at their respective optimal tolerances, 33% and 34%. This figure shows considerable variation in frequency composition of segments in cancerous breasts compared to healthy breasts. This clear, observable difference supports the use of this diagnostic segmentation methodology, demonstrating how tissue properties in a healthy breast tend to be more similar, as expected. The large variation in average frequencies of interest for both cancerous and healthy breasts in **Figure 6** further demonstrates breast properties are unique, and vary even between breasts of the same women, showing set diagnostic thresholds or comparison between breasts is likely to result in inaccurate diagnosis and poor overall performance.

Of particular interest is the varying performance of configurations 1 and 6 in diagnosing specific subjects. Configuration 6 was the optimal configuration and resulted in two false negatives, P10 and P13 (85% sensitivity), and three false positives, P5, P11 and P12 (77% specificity), already meeting Criteria #2 for a highly sensitive diagnostic algorithm in **Table 2**. In contrast, configuration 1 resulted in three false negatives, P1, P6 and P11 (77% sensitivity) and three positives, P5, P12 and P14L (77% specificity). Thus, while false positives P5 and P12 were diagnosed incorrectly in both configurations, all false negative diagnoses were diagnosed correctly in one of the two configurations.

Differing diagnostic success for different subjects in each configuration shows tumor location potentially affects the efficacy of diagnosis in certain configurations. Fitzjohn et al. suggests tumor presence can often affect the properties of segments either side (30), and, as such, using these segments as the control may result in less distinguishable properties compared to a segment far from the tumor, where a greater difference will result in a more prominent cancer diagnostic.

Configurations 1 and 6 are situated on opposite sides of the breast and, when combined, result in all cancer being diagnosed in at least one segment. Therefore, there is potential for two opposite segments to be used to ensure diagnosis of all cancers. If diagnosis in either configuration was to result in positive diagnosis, the diagnostic result would be zero false negatives (100% sensitivity) and four false positives (69% specificity), meeting Criteria #2 in **Table 2**, with perfect sensitivity and still acceptable specificity (>65%). This outcome shows a significant diagnostic using a computationally efficient algorithm. Further metrics could be designed to potentially combine with other DIET diagnostic methods and reduce false positive results.

Combining results may improve sensitivity but increase false positives and unnecessary biopsies, which already impact almost 20% of women (59). Clinical breast examination or other breast screening tools may also be utilised to ensure unnecessary breast biopsies are reduced. In particular, positive DIET results could be immediately followed up with skilled manual palpation or ultrasound to reduce this risk and reduce the time taken to women receiving diagnostic outcomes and consequent treatment.

**Figure 7** shows different patient, breast and tumor information for the false negatives in both configurations 1 and 6. It shows all false negatives are less than 20 mm, which is associated with lower stage cancer (60) and expectedly considered more difficult to diagnose. Two of the false negatives are the two smallest tumors in this cohort, at 7mm. Most importantly, all cancers are detected in one of the configurations, showing the capability of detecting both 7mm tumors, depending on breast configuration.

**Figure 7** also shows all five false negatives across each configuration are five of the six smallest tumor to volume ratios, expected to be more difficult to diagnose in methods comparing average breast segment properties. Additionally, P10, P11 and P13 have unknown tumor depth, which, if deep, could also cause diagnostic issues (30). **Figure 8** shows patient age and breast volume for false negative and false positive cases. False negative results occur at a range of breast sizes and average ages for this cohort. More importantly, the true positives are patients with varied ages and breast sizes, suggesting there is no diagnostic limitation of age or volume related breast properties for this algorithm, showing an equitable diagnostic result.

**Figure 8** also shows cancer found in two of the youngest women diagnosed correctly, which is a significant result, given mammography often performs worse in young women, who tend to have higher breast density consisting of more glandular tissue, which can mask the presence of a tumor (52, 61). False positives in **Figure 8** tend to occur in smaller breasts, perhaps where differing breast structure has a more magnified effect due to smaller segments overall. Thus, adjusting the number of z-bands or segments used based on breast size could potentially reduce false positives.

False positive results could be the result of some complex internal tissue differences around the breast causing distinguishing properties when using this breast segmentation methodology. However, it is important to note a missed diagnosis in mammography should not be ruled out. Specifically, the false positive patients all have smaller breasts, also associated with potentially increased breast density, and thus, worse outcomes in mammography (62). Furthermore, Patients P5 and P14L are the two youngest patients in this cohort. Generally, breast density decreases with increasing age (52, 61), and, as such, these false positive patients may have dense breasts, causing known issues for diagnosis using mammography, as dense tissue masks the presence of a tumor (63). Unfortunately, no follow up information is available with this data set regarding each patient’s outcomes and consequent screenings. Test information is given as a one-off and, as such, we may never know this outcome.

Important to note is the right breast in patient P14R, which was correctly diagnosed as healthy in both configurations. This subject was originally diagnosed with cancer in mammography, which was later proven to be healthy tissue. Successful identification of this breast using this method shows an instance where DIET diagnostic capabilities were able to out perform mammography. This outcome helps prove implementation of DIET into the breast screening system could potentially improve overall diagnostic accuracy. Patient P6’s right breast was also correctly identified as healthy in both configurations, despite having a non-malignant cyst, again showing the potential for DIET algorithms to distinguish between tumors and non-malignant lesions based on tissue stiffness.

With current limited clinical data, the method is primarily focused on the detection of tumor presence as a binary labelling problem. Detecting the exact location and depth of tumor for surgery and treatment purpose would require a much larger cohort of data to build its non-linear correlation to tissue properties and motion dynamics to avoid over-fitting issues. However, the current result did imply the frequency of interest for cancerous segments presented a notable contrast of response to healthy segments, which could be used to provide a preliminary estimation of location. Therefore, the benefit of the method is the comfort and ease of screening and the automated results, which keeps running costs low, increases breast screening equity and encourages screening participation. While location around the breast segment could be achieved with this algorithm, further clinical breast exam, ultrasound, mammography or MRI would be recommended for confirming the exact location and depth.

### Algorithm robustness and configuration selection

**Figures 10**, **11** show the bootstrapped ROC curves for all breast configurations following bootstrapping with 50 breasts selected with replacement and a repetition of 200 trials. **Table 3** shows the AUC, optimal sensitivity and specificity, as well as sensitivity and specificity for criteria in **Table 2** for each configuration. Bold values show configurations which meet the accuracy and AUC criteria.

Nine configurations (1, 4–7, 9–12) met the criteria for AUC over 0.73, which shows the algorithm is fairly robust to configuration selection, although some configurations are clearly more optimal. Four configurations (1, 6, 9, 11) met criteria for specificity greater than 65% when a highly sensitive (80%) diagnosis is achieved (Criteria #2). These configurations and an additional configuration 5 also met criteria for sensitivity at least 60% when specificity is similar to mammography (80%) (Criteria #1).

In general, the most optimal breast configurations occurred towards the top and bottom of the breast. The increased diagnostic quality of these segments might be attributed to the natural way the breast hangs. Breast tissue structure is inhomogeneous and its complex structure and fibrous frame continually change with age due to effects, such as gravity (58, 64). The DIET set up may also distort surface motion in the natural hanging position due to pre-tensioning and pre-compression of surface tissue. It is possible using control segments at the top and bottom of the breast result in maximum surface tension, including the presence of suspensory (cooper’s) ligaments (64) and, thus provide the truest steady state response for frequency analysis with minimum non-homogeneous tissue mechanics. Thus, these configurations result in optimal diagnosis for this frequency dependent diagnostic algorithm.

The optimal configuration (6) well exceeded performance criteria in **Table 2** with optimal AUC at 0.85, optimal sensitivity and specificity of 81% and 75%. Sensitivity was 74% when specificity was similar to mammography (Criteria #1) and specificity was 76% when a highly sensitive diagnostic was achieved (Criteria #2). As mentioned, AUC of 0.85 is considered excellent (>0.8) (41), and not only well exceeds criteria (0.73), but exceeds all AUC values identified in studies on mammography (0.54-0.84) (12, 31, 40, 42–46).

As shown in **Figures 6**, **7**, a combination of configuration 1 and 6 could result in perfect sensitivity at 100% and specificity of 69% exceeding criteria for a highly sensitive diagnostic (>65% in Criteria #2). This highly successful diagnostic outcome proves diagnostic efficacy using DIET can be achieved, supporting further research and investment in this technology.

### Assessment against diagnostic criteria

All diagnostic criteria outlined in **Table 2** were met or exceeded by the diagnostic method described in this paper. **Table 4** shows optimal AUC well exceeds criteria at 0.85 (>0.73), and both accuracy criteria are well exceeded with Criteria #1 sensitivity at 74% (>60%) and Criteria #2 specificity at 76% (>65%). Furthermore, both 7 mm tumors were able to be correctly diagnosed in one of two configurations analysed, showing diagnosis of tumors, below theaverage tumor size detected by mammography at 10~14 mm (27, 65).

## Limitations

The main limitation of this study is the limited clinical data available. This study presents results based 26 breasts analysed from 14 patients from a limited technical trial. Increased funding would enable more clinical trials and thus increased data, greatly improving validation of results and allowing for deep learning techniques to be utilised. However, the data in this cohort includes patients with a range of breast sizes and varying tumor sizes and depths (**Table 1**), which demonstrate the success and potential of diagnostic algorithm across a varied cohort. Lack of displacement data for some patients at relevant input frequencies resulted in exclusion of one breast from each of Patient P4 and P13 in the analysis presented in this paper. All excluded breasts were healthy, so assessing the ability of this algorithm to diagnose cancerous breasts was not affected.

It should be noted, machine learning methods have been successfully applied for identification, diagnostic and analysis of medical data (66, 67). However, training the models of machine learning and deep neural networks normally require a very large cohort of labelled data in the medical field, which is not available in this case. Moreover, very detailed patient demographics and extra examinations might be needed to construct efficient input features to the training models, which may not necessarily be practical for a quick and equitable screening implementation such as *via* the DIET system. Therefore, a physics-based method and approach is considered to be more appropriate for this technology and at this time than machine learning methods, given the currently limited data for the DIET screening system. In future, such approaches could provide significant new diagnostic approaches.

Another limitation identified was the fitting of the ROC curve equation *y=*1*-e^-ax^* for some configurations. This equation was chosen at it is able to represent the linear (50:50 chance) and, with a very large exponent, the perfect square ROC curve,as well as all squares in between. However, it is restricted by the use of only one parameter (a), meaning there is a fixed relationship between sensitivity and specificity. Increasing the number of fitting parameters, such as *y=*1*-e^-a^* ^(^ *^x-b^* ^)^+*c*, would potentially over fit the data and may result in not meeting the 0 to 1 bounds of the ROC curve.

For instance when comparing configuration 2 in **Figure 10** and configuration 8 in **Figure 11**, both have a similar bootstrapped ROC curve shape. **Table 3** show identical AUC, optimal sensitivity and specificity and criteria values. Despite these similarities, the trend of the curve differs. Configuration 2 tends towards higher specificity, whereas configuration 8 appears more sensitive. These subtle differences are not captured by the fitting of equation *y=*1*-e^-ax^* with just one parameter. This issue is a limitation of this fit and development of this ROC curve model may more successfully capture the trade-off of sensitivity and specificity in some configurations. In general, the equation was successful in comparing configurations and most curves captured the general trend of points.

## Conclusions

This paper presents a computationally efficient diagnostic algorithm, which meets identified criteria for comparable accuracy to mammography and the ability to provide a highly sensitive diagnostic in breast screening. Three-dimensional plots showing response frequencies demonstrate how cancerous breasts exhibit higher and more varied frequencies of interest. An unbiased diagnostic was developed using a segmentation methodology, comparing second dominant frequencies in various breast segments, with similar frequencies expected in healthy breasts and more distinguishable differences indicating potential tumors. This method allowed for each breast to be diagnosed independently, removing issues of highly variable breast properties on diagnostic success.

Patient data at frequencies analysed was available for a total of 26 breasts (13 healthy and 13 cancerous) from 14 patients. An optimal breast configuration and diagnostic tolerance resulted in 85% sensitivity and 77% specificity. Using two configurations on either side of the breast demonstrated how sensitivity could be increased to 100% with only one additional false positive (specificity 69%), still meeting criteria for a highly sensitive diagnostic with manageable false positives. All diagnostic criteria were well exceeded showing potential for diagnosis using DIET to exceed diagnostic accuracy of mammography, including one breast correctly identified using this method, which was a false positive in mammography. ROC curve AUC exceeded all identified AUC values for mammography at 0.85 (0.54-0.84) and when specificity was similar to mammography (80%) sensitivity far exceeded it at 74% (>60%). This study provides an unbiased, fully automated diagnostic algorithm capable of detecting all tumors in this cohort, with manageable false positives, proving the diagnostic potential of the DIET technology, as a breast screening tool with many benefits.

## Data availability statement

The data analyzed in this study is subject to the following licenses/restrictions: Clinical data can be requested from Tiromedical, NZ. Requests to access these datasets should be directed to jessica.fitzjohn@pg.canterbury.ac.nz.

## Ethics statement

The studies involving human participants were reviewed and approved by New Zealand Health and disability ethics committee (URA/08/08/059). The patients/participants provided their written informed consent to participate in this study.

## Author contributions

JF did the conceptualisation, analysis and writing, CZ and JGC provided conceptualisation, aided analysis and revised writing. All authors contributed to the article and approved the submitted version.

## Funding

The authors acknowledge UC Doctoral Scholarship funding.

## Conflict of interest

The authors declare that the research was conducted in the absence of any commercial or financial relationships that could be construed as a potential conflict of interest.

## Publisher’s note

All claims expressed in this article are solely those of the authors and do not necessarily represent those of their affiliated organizations, or those of the publisher, the editors and the reviewers. Any product that may be evaluated in this article, or claim that may be made by its manufacturer, is not guaranteed or endorsed by the publisher.
